# The Therapeutic Effect of Tapinarof on In Vitro Cutaneous Lupus-like Keratinocyte Model

**DOI:** 10.3390/ijms27135828

**Published:** 2026-06-28

**Authors:** Yoko Kuba-Fuyuno, Gaku Tsuji, Makiko Kido-Nakahara, Kazuhiko Yamamura, Sawako Sakai, Takeshi Nakahara

**Affiliations:** 1 Department of Dermatology, Graduate School of Medical Sciences, Kyushu University, 3-1-1 Maidashi, Higashiku, Fukuoka 812-8582, Japan; tsuji.gaku.893@m.kyushu-u.ac.jp (G.T.); nakahara.makiko.542@m.kyushu-u.ac.jp (M.K.-N.); sawako.sakai.400@m.kyushu-u.ac.jp (S.S.); nakahara.takeshi.930@m.kyushu-u.ac.jp (T.N.); 2 Research and Clinical Center for Yusho and Dioxin, Kyushu University Hospital, 3-1-1 Maidashi, Higashiku, Fukuoka 812-8582, Japan

**Keywords:** cutaneous lupus erythematosus, aryl hydrocarbon receptor, tapinarof, interferon

## Abstract

Cutaneous lupus erythematosus (CLE) is an autoimmune disease in which type I interferon (IFN) has been well-established as a central pathogenic factor. It presents with diverse cutaneous manifestations that markedly impair patients’ quality of life. Therapeutic options for CLE remain limited and are often insufficient, highlighting the need for novel treatment strategies. The aryl hydrocarbon receptor (AHR) is a transcription factor that modulates immune responses and has recently attracted attention as a potential therapeutic target for autoimmune diseases. We previously confirmed that treatment of normal human epidermal keratinocytes with IFNα and Poly I:C upregulated the expression of the CLE-like inflammatory cytokines IFNκ, CXCL10, and IL6, as well as the B-cell activating factor. In the present study, we demonstrated by quantitative PCR and Western blotting that pretreatment with the AHR agonist tapinarof suppressed the upregulation of IFNκ and CXCL10. Mechanistically, we demonstrated that tapinarof suppresses IFNκ expression by inhibiting STAT1 phosphorylation in an AHR-dependent manner, thereby attenuating the nuclear translocation of ISGF3. Overall, we show that tapinarof suppresses CLE-like inflammation in human keratinocytes in an AHR-dependent manner, suggesting that it may represent a novel therapeutic agent for CLE.

## 1. Introduction

Lupus erythematosus is a chronic autoimmune disease that comprises a variety of clinical manifestations. Cutaneous lupus erythematosus (CLE) occurs in 70% of all patients with systemic lupus erythematosus (SLE) [1]. CLE is characterized by a wide spectrum of skin manifestations and substantially impairs patients’ quality of life. Although hydroxychloroquine has expanded treatment options, it carries the risks of retinal damage and cardiotoxicity. Other treatments are limited to topical steroids and light avoidance, which are insufficient, thus there is a need to develop new, safer drugs for this condition [2].

The expression of type I interferon (IFN)-regulated proinflammatory cytokines is a hallmark of CLE skin lesions [1], and interferon-alpha (IFNα) is the primary therapeutic target for CLE [3]. Poly I:C is a synthetic nucleic acid, which is reported to exacerbate lupus in model mice [4]. Previously, we proposed a CLE-like keratinocyte model in vitro reflecting local lesional conditions using IFNα and Poly I:C [5]. In this model, we showed that stimulation of human keratinocytes by IFNα and Poly I:C induced CLE-like inflammatory cytokines (such as IFNκ, CXCL10, IL6, IFNλ-receptor, and IL18), the expression of B-cell activating factor (BAFF) and BAFF receptor, apoptosis signals, and apoptotic cells [5].

The aryl hydrocarbon receptor (AHR) is essential for maintaining homeostasis in skin and tissues, including with regard to the barrier function of epidermal cells, and has been reported to be an important therapeutic target for autoimmune diseases [6]. Tapinarof, an AHR agonist and a therapeutic AHR-modulating agent (TAMA), was reported to suppress inflammation in psoriasis and atopic dermatitis in an AHR-dependent manner [7], and has been clinically proven to be effective for psoriasis and atopic dermatitis in daily clinical practice [8]. Meanwhile, it was reported that tapinarof’s inhibition of JAK-STAT phosphorylation in a mouse model of lupus suppressed immune complex deposition and alleviated splenomegaly, lymphadenopathy, and nephropathy [9]. In addition, Gangs et al. reported that discoid lupus erythematosus (DLE) was successfully treated with topical tapinarof [10]. Taken together, these findings suggest that tapinarof may be effective for CLE; however, the underlying mechanisms remain unclear, and no studies have yet investigated this possibility.

This study investigates the effects of tapinarof, an AHR ligand, in an in vitro CLE keratinocyte model using normal human epidermal keratinocytes (NHEKs) derived from female donors within the young to middle-aged age range commonly affected by SLE, through comparison with skin biopsy samples from acute CLE (ACLE) patients. It also assesses the potential of tapinarof as a novel therapeutic candidate for the treatment of CLE.

## 2. Results

### 2.1. Tapinarof Did Not Affect the Viability of Keratinocytes

Tapinarof at a concentration of 500 nM did not affect the viability of NHEKs, consistent with a previous report (Supplementary Figure S1) [11].

### 2.2. Tapinarof Suppressed the Expression of IFNκ, CXCL10, IL6, and BAFF in a CLE-like Keratinocyte Model In Vitro Under Conditions That Induced AHR Expression

**Figure 1:**
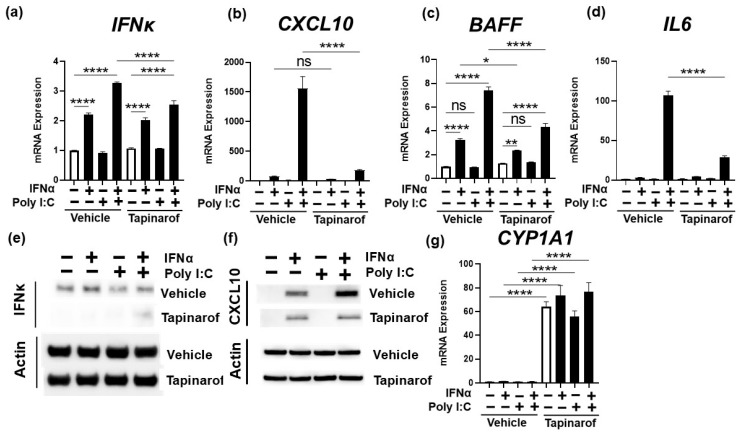
NHEKs were pretreated with tapinarof (500 nM) for 24 h and subsequently stimulated with IFNα and Poly I:C for an additional 24 h. (**a**–**d**,**g**) The mRNA expression of IFNκ, CXCL10, BAFF, IL6, and CYP1A1 was analyzed by qRT-PCR. The reproducibility of the results was confirmed in three independent experiments, each yielding the same overall trend and conclusion. Data are expressed as mean ± S.E.M.; *n* = 3 for each group. Statistically significant differences between the expression of control and tapinarof-treated NHEKs are presented: * *p* < 0.05; ** *p* < 0.01; **** *p* < 0.0001; ns, not significant. White bars represent control conditions (untreated and tapinarof alone), whereas black bars represent the corresponding conditions with inflammatory stimulation. (**e**,**f**) The protein expression of IFNκ and CXCL10 was examined by Western blotting.

First, we examined whether tapinarof downregulates CLE-like inflammation and B-cell induction in NHEKs. NHEKs were pretreated for 24 h with tapinarof (500 nM), followed by treatment with IFNα (15 ng/mL) and Poly I:C (15 ng/mL) for 24 h. The results showed that tapinarof treatment downregulated IFNκ, CXCL10, BAFF, and IL6 induced by IFNα and Poly I:C, and upregulated CYP1A1, an indicator of AHR activation (Figure 1a–g).

**Figure 2:**
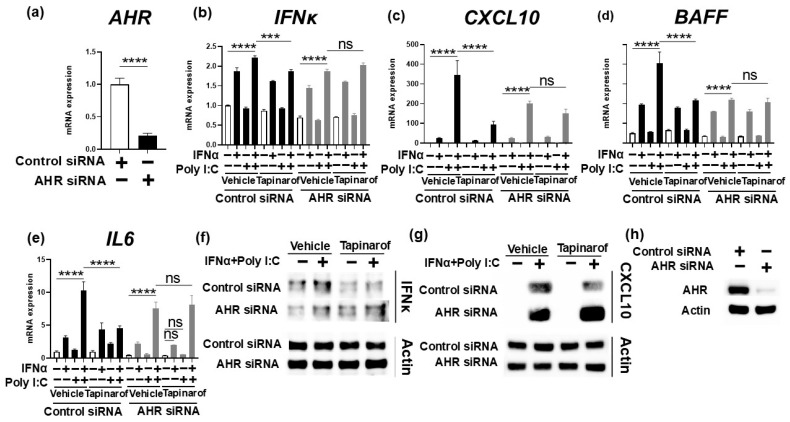
NHEKs were transfected with either control siRNA (control siRNA) or siRNA against AHR (AHR siRNA) for 48 h. (**a**) Successful knockdown of AHR was confirmed by qRT-PCR analysis. Transfected NHEKs were subsequently treated with IFNα and Poly I:C for 24 h to establish a CLE-like keratinocyte model with or without tapinarof (500 nM). White bars indicate the control siRNA group, and black bars indicate the AHR siRNA group. (**b**–**e**) The mRNA levels of IFNκ, CXCL10, BAFF, and IL6 were analyzed by qRT-PCR. White bars represent the untreated control and the tapinarof-alone control. Black bars represent the inflammatory stimulation groups transfected with control siRNA. Gray bars represent the inflammatory stimulation groups transfected with AHR siRNA. Data are expressed as mean ± S.E.M.; *n* = 3 for each group. Statistically significant differences between the expression of control and tapinarof-treated NHEKs are presented: *** *p* < 0.001; **** *p* < 0.0001; ns, not significant. (**f**,**g**) The protein levels of IFNκ and CXCL10 were analyzed by Western blotting. (**h**) Successful knockdown of AHR was confirmed by Western blotting.

To examine whether these anti-inflammatory effects and B-cell-inducing abilities of tapinarof are dependent on AHR, NHEKs were transfected with either control siRNA (control siRNA) or siRNA against AHR (AHR siRNA). The NHEKs were subsequently treated with IFNα and Poly I:C for 48 h with or without tapinarof (500 nM). The results indicated that the suppression of IFNκ, CXCL10, BAFF, and IL6 mRNA and IFNκ and CXCL10 protein expression by tapinarof was counteracted by AHR knockdown (Figure 2a–h). We thus revealed that tapinarof treatment downregulated IFNκ, CXCL10, BAFF, and IL6 induced in CLE-like keratinocytes in vitro via AHR.

### 2.3. IRF9 and pSTAT1 Expression Was Elevated in the Skin of ACLE Patients

**Figure 3:**
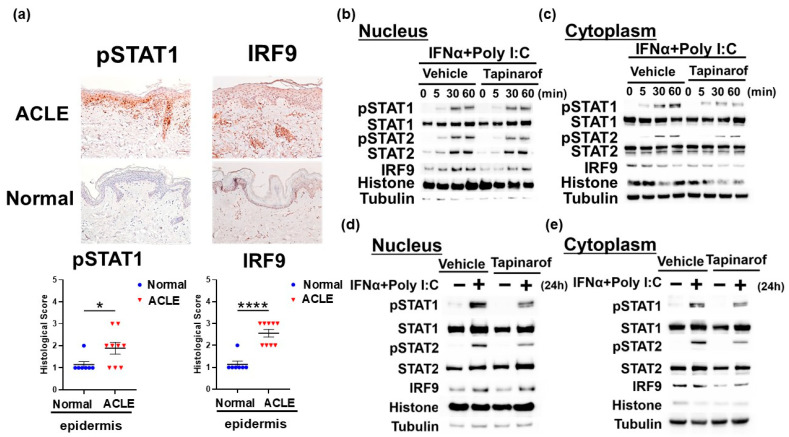
IRF9 and pSTAT1 expression was elevated in the skin of ACLE patients, but tapinarof suppressed the expression of pSTAT1, pSTAT2, and IRF9. (**a**) We compared the characteristics of ACLE in the skin of SLE patients (*n* = 7) and normal controls (*n* = 7) by analyzing the expression of pSTAT1 and IRF9 in the epidermis immunohistochemically. * *p* < 0.05; **** *p* < 0.0001. (**b**–**e**) Nuclear and cytoplasmic fractions were isolated, and the protein levels of pSTAT1, pSTAT2, and IRF9 were analyzed by Western blotting to assess their nuclear translocation.

It is reported that interferon regulatory factor 9 (IRF9) and STAT1 are involved in the regulation of interferon-stimulated gene (ISG) expression in SLE [12,13]. We thus compared the characteristics of ACLE in the skin of SLE patients (*n*  =  7) and normal controls (*n*  =  7) by analyzing the expression of pSTAT1 and IRF9 in the epidermis immunohistochemically (Table S1). All patients met the 1997 American College of Rheumatology revised criteria for the classification of SLE. All protocols were approved by the Institutional Review Board of the University of Kyushu Hospital. As shown in Figure 3a, pSTAT1 and IRF9 expression was significantly increased in the epidermis of ACLE-affected skin.

### 2.4. IFNα and Poly I:C Increased the Expression of IFNκ via pSTAT1-pSTAT2-IRF9 in Human Keratinocytes, and Tapinarof Suppressed the Expression of pSTAT1, pSTAT2, and IRF9 via AHR

IRF9 joins the pSTAT1-pSTAT2 heterodimer to complete the transcription factor pSTAT1-pSTAT2-IRF9 complex (ISGF3), which translocates to the nucleus and binds to interferon-stimulated response elements (ISREs) in ISG promoters [13]. Therefore, we investigated whether the suppression of IFNκ by tapinarof depends on ISGF3 in human keratinocytes. To this end, we examined the effect of tapinarof on the downregulation of the expression of pSTAT1-pSTAT2-IRF9 in NHEKs. To clarify the intracellular localization, we evaluated the protein expression of pSTAT1, pSTAT2, and IRF9 by Western blotting separately for the nucleus and cytoplasm for 60 min and 24 h. First, we examined whether IFNα and Poly I:C induced ISGF3 in keratinocytes. Consistent with our hypothesis, IFNα and Poly I:C induced the protein expression of IRF9, pSTAT1, and pSTAT2 in keratinocytes for 60 min and 24 h. Furthermore, tapinarof inhibited the phosphorylation of STAT1 and STAT2 in both the nucleus and cytoplasm for 60 min and 24 h. Tapinarof also suppressed both the nuclear translocation of IRF9 for 60 min and the expression of IRF9 in the cytoplasm for 24 h (Figure 3b–e). Quantification by ImageJ software (version 1.54i 03 March 2024, National Institutes of Health, Bethesda, MD, USA) demonstrated that tapinarof-mediated suppression of STAT1 phosphorylation and IRF9 expression peaked at 30 min. The diminished suppression observed at 60 min may indicate the presence of a feedback mechanism or cyclical regulation of signaling activity. At 24 h, when setting the findings for the IFNα and Poly I:C-treated group to 100%, nuclear accumulation of pSTAT1 and IRF9 was reduced by tapinarof by 39.1% and 12.9%, respectively. In the cytoplasmic fraction, the corresponding decreases were 40.1% and 61.7%, respectively (Supplementary Figures S2 and S3).

**Figure 4:**
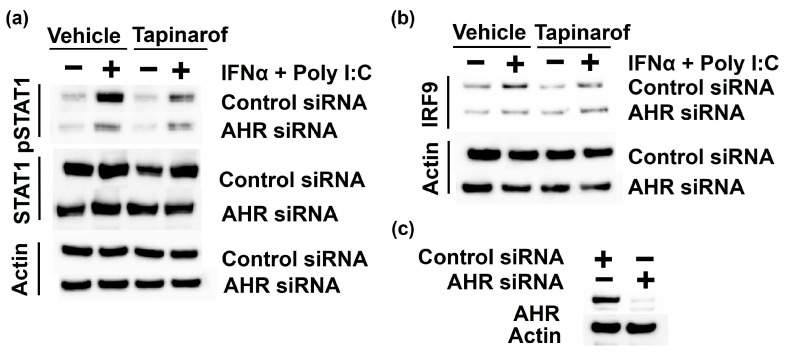
Tapinarof suppressed the expression of pSTAT1, pSTAT2, and IRF9 via AHR. (**a**,**b**) NHEKs transfected with siRNA against AHR were treated with IFNα and Poly I:C for 24 h, with or without tapinarof (500 nM), and protein expression of pSTAT1, pSTAT2, and IRF9 was analyzed by Western blotting. (**c**) Successful knockdown of AHR was confirmed by Western blotting.

Next, we examined whether tapinarof inhibits pSTAT1-pSTAT2-IRF9 in an AHR-dependent manner. NHEKs transfected with siRNA against AHR were treated with IFNα and Poly I:C for 24 h, with or without tapinarof (500 nM), and the protein expression of pSTAT1, pSTAT2, and IRF9 was analyzed by Western blotting. The results revealed that the downregulation of pSTAT1, pSTAT2, and IRF9 induced by tapinarof tended to be abolished by AHR knockdown (Figure 4a–c). The above findings demonstrate that tapinarof suppresses the expression of pSTAT1, pSTAT2, and IRF9 via AHR.

### 2.5. Tapinarof Suppressed the Expression of IRF9 and IFNκ via STAT1 in Human Keratinocytes

**Figure 5:**
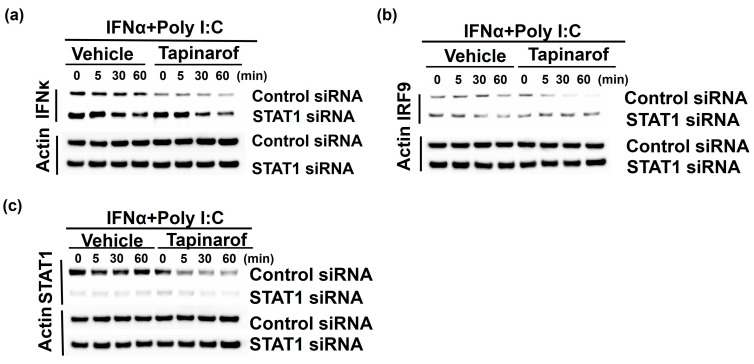
Tapinarof suppressed the expression of IFNκ and IRF9 via STAT1 in human keratinocytes. (**a**,**b**) NHEKs transfected with siRNA against STAT1 were treated with IFNα and Poly I:C for 24 h, with or without tapinarof (500 nM), and the protein expression of IFNκ and IRF9 was analyzed by Western blotting. (**c**) Successful knockdown of STAT1 was confirmed by Western blotting.

We also investigated whether the effects of tapinarof of suppressing IRF9 expression in an AHR-dependent manner and reducing IFNκ expression were dependent on STAT1. NHEKs transfected with siRNA against STAT1 were treated with IFNα and Poly I:C for 24 h, with or without tapinarof (500 nM), and the protein expression of IFNκ, IRF9, and STAT1 was analyzed by Western blotting. As shown in Figure 5, the downregulation of IFNκ and IRF9 induced by tapinarof was abolished by STAT1 knockdown in keratinocytes.

## 3. Discussion

CLE is a chronic relapsing inflammatory skin disease, in which long-term remission is rare and the need for ongoing treatment is common [1,3]. For the treatment of CLE, topical options have long been limited to topical corticosteroids and calcineurin inhibitors [1]. Although systemic glucocorticoids and hydroxychloroquine are also widely used for CLE, the former typically exert a broad immunosuppressive effect and the latter carries the risk of retinopathy while also varying in effectiveness from patient to patient [1,3]. The availability of targeted therapies has improved the management of SLE, but there is still an unmet need for safe, long-term treatments capable of achieving sustained disease control.

Type I IFNs have a crucial role in the pathogenesis of CLE [1,2]. They are produced by recruited inflammatory cells and by the epidermis itself (IFNκ) and play important roles in a range of autoimmune and inflammatory skin diseases [12]. Sarkar et al. reported that IFNα and IFNκ are the two IFNs whose levels are significantly increased in CLE-affected skin [14]. Recently, it has been reported that IFNκ is highly expressed not only in the skin of SLE patients, but also in the skin of patients at high risk of developing SLE, and that UV irradiation further increases IFNκ expression. These findings suggest that the skin is not merely a target of immune-mediated inflammation, but may actively drive local infection responses via keratinocyte-derived IFNκ [15]. In the current study, tapinarof downregulated the expression of IFNκ, CXCL10, IL6, and BAFF in a CLE-like keratinocyte model. Notably, our results showed that the effects of tapinarof on suppressing IFNκ, CXCL10, IL6, and B-cell induction were dependent on AHR. To the best of our knowledge, no previous studies have demonstrated the inhibitory effect of tapinarof on IFNκ in epidermal keratinocytes.

AHR is a ligand-activated transcription factor that has been extensively studied because of its involvement in metabolizing environmental toxins [16,17]. Although it is reported that DLE was successfully treated with topical tapinarof, the mechanism underlying this has not yet been elucidated [10]. AHR is a key regulator of immune responses and has been implicated in the pathogenesis of various autoimmune diseases, including multiple sclerosis, rheumatoid arthritis, inflammatory bowel disease, and SLE [6]. Several studies have also highlighted the potential of AHR as a therapeutic target for SLE, based on its immunomodulatory roles [6,18,19]. Beyond its immunomodulatory functions, environmental factors such as ultraviolet radiation and cigarette smoke are known to aggravate SLE. As AHR serves as a key interface between environmental signals and immune responses, Wu et al. have suggested that it may represent a therapeutic target for SLE [6].

In this study, we demonstrated that tapinarof suppresses CLE-like inflammation by inhibiting STAT1 phosphorylation, similarly via the AHR pathway. Zhang et al. reported that tapinarof ameliorates lupus autoimmunity via regulation of the JAK-STAT signaling pathway. This pathway plays a critical role in inflammation, immune responses, and autoimmune diseases, including SLE [9,20]. Type I IFNs bind to the IFN-α/β receptor and initiate an amplification loop that activates the receptor-associated kinases Tyk2 and JAK1. This leads to the recruitment and phosphorylation of STAT1 and STAT2. IRF9 then associates with the STAT1/STAT2 heterodimer to form the ISGF3 complex, which binds to ISREs in DNA and induces the transcription of ISGs [20]. In another study, Deng et al. analyzed ISG expression profiles and their regulatory pathways in SLE and lupus nephritis, identifying IRF9 and STAT1 as potential key regulators of ISG expression [21]. We found that tapinarof suppressed the phosphorylation of STAT1 and the expression of IRF9. Previous studies reported that AHR regulates STAT1 activation [22,23] and that the interaction between AHR and STAT1 [24] and tryptophan metabolism activates AHR, leading to inhibition of the JAK-STAT1 signaling pathway and consequent reductions in the secretion of CXCL9 and CXCL10 [25]. Furthermore, in this study, the tapinarof-induced inhibition of STAT1 phosphorylation and suppression of IRF9 expression were abrogated by the knockdown of AHR. These findings demonstrate that tapinarof suppressed IFNκ expression by inhibiting STAT1 phosphorylation via AHR, thereby suppressing the expression and nuclear translocation of IRF9.

Under the conditions employed in this study, AHR activation suppressed the expression of CLE-like inflammation in keratinocytes. As these findings were obtained in the present in vitro experimental system under highly limited conditions, there is a need for further validation via in vivo and clinical investigations in order to validate them.

This study revealed that tapinarof suppresses STAT1 phosphorylation by inhibiting AHR activation, thereby inhibiting ISGF3 nuclear translocation and suppressing IFNκ expression. Tapinarof may thus be useful for treating CLE and warrants further investigation.

## 4. Materials and Methods

### 4.1. Cell Viability Assay

NHEKs were seeded at a density of 1 × 10^4^ cells per well in 96-well plates. Cell viability was measured using a CCK-8 assay. Each condition was tested in five technical replicates (quintuplicate wells), and the entire experiment was independently repeated three times. All NHEKs used in this study were derived from young to middle-aged female donors, corresponding to the age commonly affected by CLE.

### 4.2. Reagents

Tapinarof (MedChemExpress, Monmouth Junction, NJ, USA) was dissolved in dimethyl sulfoxide (DMSO; Nacalai Tesque, Kyoto, Japan) and stored at −80 °C until used in the experiments. Recombinant Human Interferon Alpha A (Alpha2a) was purchased from PBL Assay Science (Piscataway, NJ, USA), and synthetic dsRNA of Poly I:C HMW was purchased from InvivoGen (San Diego, CA, USA). The antibodies used for Western blotting and immunohistochemistry are shown in Table S2.

### 4.3. Cell Culture

NHEKs obtained from Lonza (Basel, Switzerland) were grown in culture dishes at 37 °C in 5% CO_2_. The NHEKs were cultured in serum-free keratinocyte growth medium (Lonza) supplemented with bovine pituitary extract, recombinant epidermal growth factor, insulin, hydrocortisone, transferrin, and epinephrine. Culture medium was replaced every 2 days. Near confluence (70–90%), cells were disaggregated with 0.25 mg/mL trypsin/0.01% ethylenediaminetetraacetic acid and subcultured. NHEKs at the second to fourth passages were used in all experiments.

Transfection of siRNAs against AHR: Small interfering RNAs (siRNAs) against AHR (AHR siRNA, s1200), STAT1 (STAT1 siRNA, s277), and non-targeting siRNA (control siRNA #2) were obtained from Ambion (Austin, TX, USA). Cells were incubated in the culture medium containing a mixture of 5 nM siRNA and HiPerFect Transfection Reagent (Qiagen, Venlo, The Netherlands) for 48 h (siRNA AHR and siRNA STAT1) and then used for further experiments.

### 4.4. In Vitro CLE Model Using NHEKs

To establish the CLE model, NHEKs were seeded into a 24-well culture plate (1 × 10^5^ cells/well) for quantitative reverse-transcription PCR (qRT-PCR) or into a 6-well culture plate (2.5 × 10^5^ cells/well) for Western blotting, depending on the experiment, and allowed to attach for 24 h. For siRNA transfection experiments followed by qRT-PCR analysis, NHEKs were seeded into 24-well culture plates at a density of 0.6 × 10^5^ cells/well and allowed to attach for 24 h before transfection. Then, the culture medium was replaced with KBMTM Gold Basal Medium, supplemented with bovine pituitary extract, insulin, transferrin, and epinephrine, and then incubated at 37 °C for another 24 h. Next, the medium was replaced with one containing 15 ng/mL Interferon Alpha (PBS) and 15 ng/mL Poly I:C (InvivoGen) and incubated at 37 °C for the indicated period.

### 4.5. Quantitative Reverse-Transcription PCR (qRT-PCR)

Total RNA was extracted from NHEKs using the RNeasy Mini Kit and RNase-Free DNase Set (Qiagen, Hilden, Germany) to eliminate contaminating genomic DNA. Reverse transcription was performed using the PrimeScript RT Reagent Kit (Takara Bio Inc., Kusatsu, Japan). qRT-PCR was conducted on a CFX Connect Real-time System (Bio-Rad, Hercules, CA, USA) using TB Green Premix Ex Taq II (Takara Bio Inc.), Thermo Scientific^TM^ SYBR TM Green qPCR Master Mix, or TaqMan^TM^ Fast Advanced Master Mix (Thermo Fisher Scientific Inc., Waltham, MA, USA). Amplification was initiated at 95 °C for 30 s as the first step, followed by 40 cycles of qRT-PCR at 95 °C for 5 s and at 60 °C for 20 s for TB Green Premix EX Taq II; at 95 °C for 2 min as the first step, followed by 40 cycles of qRT-PCR at 95 °C for 5 s and at 60 °C for 30 s for Thermo Scientific^TM^ SYBR^TM^ Green; and at 50 °C for 2 min and 95 °C for 20 s as the first step, followed by 45 cycles of qRT-PCR at 95 °C for 3 s and at 60 °C for 10 s as the second step for the TaqMan^TM^ Assay. mRNA expression was measured in triplicate and normalized to the expression levels of the housekeeping genes GAPDH and YWHAZ. The sequences of the primer pairs are shown in Table S3.

### 4.6. Western Blotting

NHEKs were washed with ice-cold PBS containing phosphatase inhibitors and incubated in lysis buffer to isolate nuclear and cytoplasmic extracts separately, using the Nuclear Extract Kit (Active Motif, Inc., Carlsbad, CA, USA), in accordance with the manufacturer’s instructions. NHEKs were washed with ice-cold PBS and incubated in lysis buffer (2 mM ethylenediaminetetraacetic acid [pH 8.0], 20 mM Na_3_VO_4_, 20 mM NaF, 1 mM phenylmethylsulfonylfluoride [PMSF], 1% Triton X-100, and RIPA buffer 10X [Nacalai Tesque Inc. Kyoto, Japan] consisting of 50 mM Tris-HCl, 150 mM NaCl, 1% NP-40, 0.5% sodium deoxycholate, and protease inhibitor cocktail) (pH 7.3) to isolate whole-cell protein lysates for Western blotting analysis. The protein concentration in the lysate was measured using a BCA Protein Assay Kit (Thermo Fisher Scientific, Rockford, IL, USA). Equal amounts of protein were dissolved in NuPAGE LDS sample buffer (Thermo Fisher Scientific) and a 10% sample reducing agent (Thermo Fisher Scientific). The lysates were heated at 70 °C for 10 min and then loaded into and subjected to electrophoresis in NuPAGE 4–12% Bis-Tris gels (Thermo Fisher Scientific) at 200 V for 25 min. The proteins were then transferred onto polyvinylidene difluoride membranes (Merck Millipore, Burlington, MA, USA), which were blocked with WesternBreeze^TM^ Blocker/Diluent (Thermo Fisher Scientific). The membranes were then probed with antibodies overnight at 4 °C. Horseradish peroxidase-conjugated anti-rabbit or anti-mouse IgG antibodies (Cell Signaling Technology, Danvers, MA, USA) served as secondary antibodies. The visualization of protein bands was accomplished with the SuperSignalWest Pico Chemiluminescent Substrate (Thermo Fisher Scientific) using the ChemiDoc touch imaging system (Bio-Rad, Hercules, CA, USA).

### 4.7. Immunohistochemistry

Samples of ACLE-affected skin and normal skin were embedded in paraffin by the conventional method and cut into 3-μm-thick sections. Antigen retrieval was performed using Heat Processor Solution pH9 (Nichirei Biosciences, Tokyo, Japan) at 100 °C for 40 min, and endogenous peroxidase was blocked by incubating the sections with 3% H_2_O_2_ (Nichirei Biosciences). The sections were treated with blocking solution for 1 h, incubated with anti-Phospho-Stat1 (Tyr701) (58D6) rabbit monoclonal antibody (Cell Signaling Technology, Inc., Danvers, MA, USA) or anti-IRF9 rabbit polyclonal antibody (GTX115401) (GeneTex, Inc., Irvine, CA, USA) for 30 min, followed by incubation with the secondary antibody, N-Histofine Simple Stain MAX-PO MULTI (Nichirei Biosciences). Immunodetection was conducted with 3,3-diaminobenzidine as the chromogen, followed by light counterstaining with hematoxylin. All SLE patients met the 1997 ACR revised criteria for the classification of SLE. All protocols were approved by the Institutional Review Board of the University of Kyushu Hospital.

### 4.8. Statistical Analysis

Statistical analysis was performed using GraphPad Prism Version 10.0.2 (GraphPad Software, San Diego, CA, USA). Unpaired Student’s *t* test or one-way analysis of variance was used to assess the results. A *p*-value of <0.05 was considered to indicate a statistically significant difference. All data are presented as the mean ± standard error of the mean (S.E.M.) from three independent experiments.

## Data Availability

The original contributions presented in this study are included in the article/Supplementary Material. Further inquiries can be directed to the corresponding author upon reasonable request.

## Supplementary Materials

The following supporting information can be downloaded at: https://www.mdpi.com/article/10.3390/ijms27135828/s1.

## Abbreviations

NHEK	Normal human epidermal keratinocyte
CLE	Cutaneous lupus erythematosus
SLE	Systemic lupus erythematosus
IFN	Interferon
AHR	Aryl hydrocarbon receptor
BAFF	B-cell activating factor
TAMA	Therapeutic AHR-modulating agent
DLE	Discoid lupus erythematosus
IRF9	Interferon regulatory factor 9
ISG	Interferon-stimulated gene
ACLE	Acute CLE
ISRE	Interferon-stimulated response element
ISGF3	Interferon-stimulated gene factor 3

## References

[1] Wenzel J. (2019). Cutaneous lupus erythematosus: New insights into pathogenesis and therapeutic strategies. Nat. Rev. Rheumatol..

[2] Vale E.C.S.D., Garcia L.C. (2023). Cutaneous lupus erythematosus: A review of etiopathogenic, clinical, diagnostic and therapeutic aspects. An. Bras. Dermatol..

[3] Niebel D., de Vos L., Fetter T., Brägelmann C., Wenzel J. (2023). Cutaneous lupus erythematosus: An update on pathogenesis and future therapeutic directions. Am. J. Clin. Dermatol..

[4] Kawato Y., Fukahori H., Nakamura K., Kubo K., Hiramitsu M., Morokata T. (2023). Development of a novel Poly(I:C)-induced murine model with accelerated lupus nephritis and examination of the therapeutic effects of mycophenolate mofetil and a cathepsin S inhibitor. Eur. J. Pharmacol..

[5] Kuba-Fuyuno Y., Kido-Nakahara M., Tsuji G., Sakai S., Nakahara T. (2024). Proposal of a cutaneous lupus erythematosus-like keratinocyte model in vitro under local conditions using interferon-alpha and Poly I:C and its use in examining the therapeutic effects of tyrosine kinase 2 inhibitor. J. Dermatol..

[6] Wu J., Pang T., Lin Z., Zhao M., Jin H. (2022). The key player in the pathogenesis of environmental influence of systemic lupus erythematosus: Aryl hydrocarbon receptor. Front. Immunol..

[7] Tsuji G., Yamamura K., Kawamura K., Kido-Nakahara M., Ito T., Nakahara T. (2023). Regulatory mechanism of the IL-33-IL-37 axis via aryl hydrocarbon receptor in atopic dermatitis and psoriasis. Int. J. Mol. Sci..

[8] Lebwohl M.G., Stein Gold L., Strober B., Papp K.A., Armstrong A.W., Bagel J., Kircik L., Ehst B., Hong H.C., Soung J. (2021). Phase 3 trials of tapinarof cream for plaque psoriasis. N. Engl. J. Med..

[9] Zhang Y., Pan Y., Zhang P., Wang F., Han Y., Li K., Jiang W., Wang J., Luan Y., Xin Q. (2023). AhR agonist tapinarof ameliorates lupus autoimmunity by suppressing Tfh cell differentiation via regulation of the JAK2-STAT3 signaling pathway. Immun. Inflamm. Dis..

[10] Garg K.S., Tjahjono L. (2024). Successful treatment of discoid lupus erythematosus with tapinarof 1% cream monotherapy. JAAD Case Rep..

[11] Vu Y.H., Hashimoto-Hachiya A., Takemura M., Yumine A., Mitamura Y., Nakahara T., Furue M., Tsuji G. (2020). IL-24 negatively regulates keratinocyte differentiation induced by tapinarof, an aryl hydrocarbon receptor modulator: Implication in the treatment of atopic dermatitis. Int. J. Mol. Sci..

[12] Hile G.A., Gudjonsson J.E., Kahlenberg J.M. (2020). The influence of interferon on healthy and diseased skin. Cytokine.

[13] Platanitis E., Demiroz D., Schneller A., Fischer K., Capelle C., Hart M., Gossenreiter T., Müller M., Novatchkova M., Decker T. (2019). A molecular switch from STAT2-IRF9 to ISGF3 underlies interferon-induced gene transcription. Nat. Commun..

[14] Sarkar M., Hile G., Tsoi L., Xing X., Liu J., Liang Y., Berthier C., Swindell W., Patrick M., Shuai S. (2018). Photosensitivity and type I IFN responses in cutaneous lupus are driven by epidermal derived interferon kappa. Ann. Rheum. Dis..

[15] Psarras A., Alase A., Antanaviciute A., Carr I.M., Md Yusof M.Y., Wittmann M., Emery P., Tsokos G.C., Vital E.M. (2020). Functionally impaired plasmacytoid dendritic cells and non-haematopoietic sources of type I interferon characterize human autoimmunity. Nat. Commun..

[16] Esser C., Bargen I., Weighardt H., Haarmann-Stemmann T., Krutmann J. (2013). Functions of the aryl hydrocarbon receptor in the skin. Semin. Immunopathol..

[17] Esser C., Rannug A. (2015). The aryl hydrocarbon receptor in barrier organ physiology, immunology, and toxicology. Pharmacol. Rev..

[18] Law C., Wacleche V.S., Cao Y., Pillai A., Sowerby J., Hancock B., Horisberger A., Bracero S., Skidanova V., Li Z. (2024). Interferon subverts an AHR-JUN axis to promote CXCL13^+^ T cells in lupus. Nature.

[19] Hanlon N., Gillan N., Neil J., Seidler K. (2024). The role of the aryl hydrocarbon receptor (AhR) in modulating intestinal ILC3s to optimise gut pathogen resistance in lupus and benefits of nutritional AhR ligands. Clin. Nutr..

[20] Flammer J.R., Dobrovolna J., Kennedy M.A., Chinenov Y., Glass C.K., Ivashkiv L.B., Rogatsky I. (2010). The type I interferon signaling pathway is a target for glucocorticoid inhibition. Mol. Cell. Biol..

[21] Deng Y., Zheng Y., Li D., Hong Q., Zhang M., Li Q., Fu B., Wu L., Wang X., Shen W. (2021). Expression characteristics of interferon-stimulated genes and possible regulatory mechanisms in lupus patients using transcriptomics analyses. EBioMedicine.

[22] Kimura A., Naka T., Nohara K., Fujii-Kuriyama Y., Kishimoto T. (2008). Aryl hydrocarbon receptor regulates Stat1 activation and participates in the development of Th17 cells. Proc. Natl. Acad. Sci. USA.

[23] Kimura A., Naka T., Nakahama T., Chinen I., Masuda K., Nohara K., Fujii-Kuriyama Y., Kishimoto T. (2009). Aryl hydrocarbon receptor in combination with Stat1 regulates LPS-induced inflammatory responses. J. Exp. Med..

[24] Ma J., Chen J., Wang H., Lu D., Liang K. (2023). AhR regulates VEGF expression by promoting STAT1 transcriptional activity, thereby affecting endothelial angiogenesis in acute limb ischemia. Chem. Biol. Interact..

[25] Liu X., Yang M., Xu P., Du M., Li S., Shi J., Li Q., Yuan J., Pang Y. (2024). Kynurenine-AhR reduces T-cell infiltration and induces a delayed T-cell immune response by suppressing the STAT1-CXCL9/CXCL10 axis in tuberculosis. Cell. Mol. Immunol..

