# Supplementary figures and images for: The Therapeutic Effect of Tapinarof on In Vitro Cutaneous Lupus-like Keratinocyte Model
Source: Int J Mol Sci. 2026 Jun 28;27(13):5828. doi: 10.3390/ijms27135828 (PMC13362408; doi:10.3390/ijms27135828)

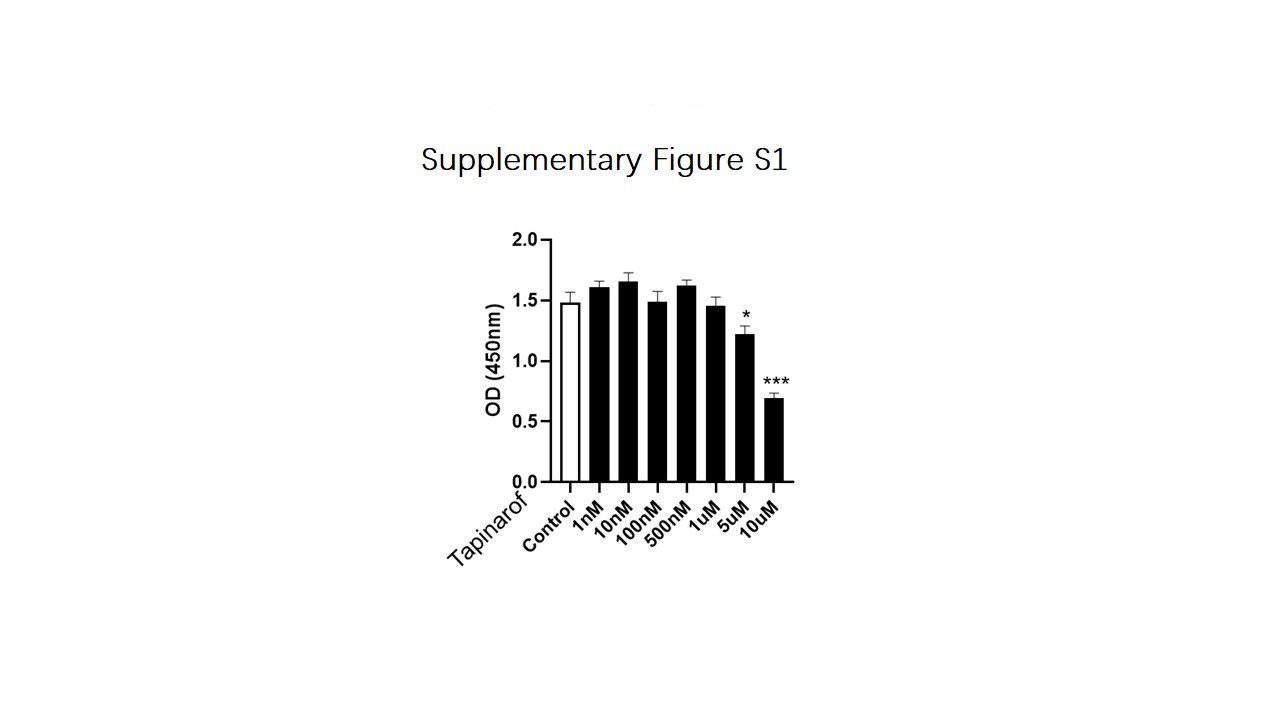

Supplement: Supplementary file 1 [file ijms-27-05828-s001.zip › Supplementary Figure S1.jpg]

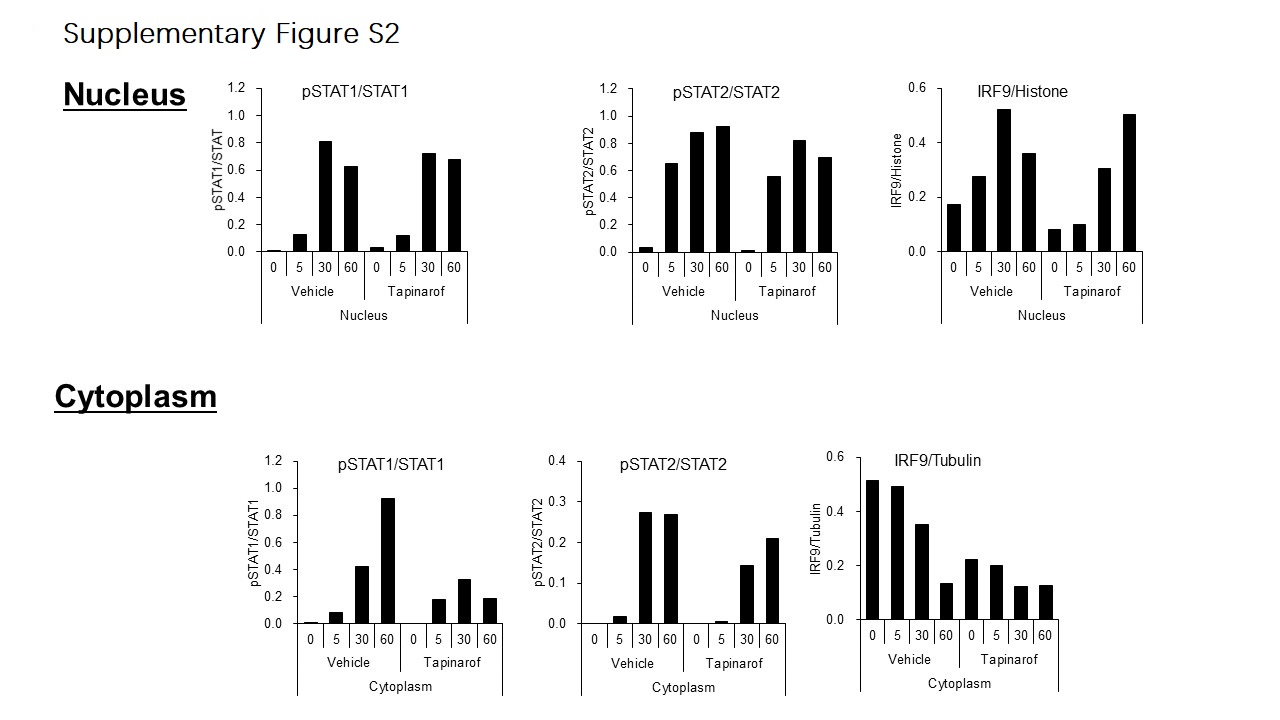

Supplement: Supplementary file 1 [file ijms-27-05828-s001.zip › Supplementary Figure S2.jpg]

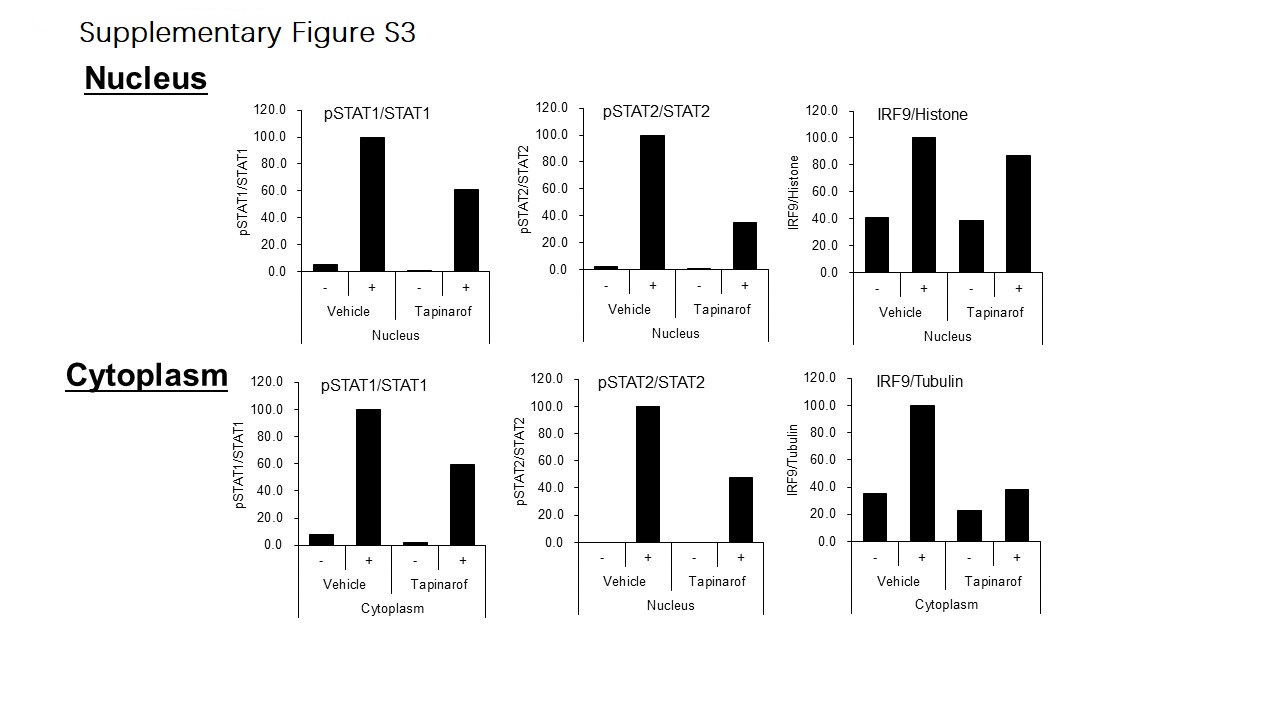

Supplement: Supplementary file 1 [file ijms-27-05828-s001.zip › Supplementary Figure S3.jpg]
